# The risk of major osteoporotic fractures with GLP-1 receptor agonists when compared to DPP-4 inhibitors: A Danish nationwide cohort study

**DOI:** 10.3389/fendo.2022.882998

**Published:** 2022-10-10

**Authors:** Zheer Kejlberg Al-Mashhadi, Rikke Viggers, Rasmus Fuglsang-Nielsen, Peter Vestergaard, Søren Gregersen, Jakob Starup-Linde

**Affiliations:** ^1^ Steno Diabetes Center Aarhus, Aarhus University Hospital, Aarhus, Denmark; ^2^ Department of Clinical Medicine, Aarhus University, Aarhus, Denmark; ^3^ Steno Diabetes Center North Jutland, Department of Endocrinology, Aalborg University Hospital, Aalborg, Denmark; ^4^ Department of Clinical Medicine, Aalborg University, Aalborg, Denmark; ^5^ Department of Endocrinology and Internal Medicine, Aarhus University Hospital, Aarhus, Denmark

**Keywords:** GLP-1, DPP-4, fracture, diabetes, bone, osteoporosis, antidiabetic, glucose-lowering drugs

## Abstract

**Background:**

Type 2 diabetes mellitus (T2D) is associated with an increased fracture risk. There is little evidence for the effects of glucagon-like peptide 1 receptor agonists (GLP-1RA) on fracture risk in T2D. We aimed to investigate the risk of major osteoporotic fractures (MOF) for treatment with GLP-1RA compared to dipeptidyl peptidase 4 inhibitors (DPP-4i) as add-on therapies to metformin.

**Methods:**

We conducted a population-based cohort study using Danish national health registries. Diagnoses were obtained from discharge diagnosis codes (ICD-10 and ICD-8-system) from the Danish National Patient Registry, and all redeemed drug prescriptions were obtained from the Danish National Prescription Registry (ATC classification system). Subjects treated with metformin in combination with either GLP-1RA or DPP-4i were enrolled from 2007 to 2018. Subjects were propensity-score matched 1:1 based on age, sex, and index date. MOF were defined as hip, vertebral, humerus, or forearm fractures. A Cox proportional hazards model was utilized to estimate hazard rate ratios (HR) for MOF, and survival curves were plotted using the Kaplan-Meier estimator. In addition, Aalen’s Additive Hazards model was applied to examine additive rather than relative hazard effects while allowing time-varying effects.

**Results:**

In total, 42,816 individuals treated with either combination were identified and included. After matching, 32,266 individuals were included in the main analysis (16,133 in each group). Median follow-up times were 642 days and 529 days in the GLP-1RA and DPP-4i group, respectively. We found a crude HR of 0.89 [0.76–1.05] for MOF with GLP-1RA compared to DPP-4i. In the fully adjusted model, we obtained an unaltered HR of 0.86 [0.73–1.03]. For the case of hip fracture, we found a crude HR of 0.68 [0.49–0.96] and a similar adjusted HR. Fracture risk was lower in the GLP-1RA group when examining higher daily doses of the medications, when allowing follow-up to continue after medication change, and when examining hip fractures, specifically. Additional subgroup- and sensitivity analyses yielded results similar to the main analysis.

**Conclusion:**

In our primary analysis, we did not observe a significantly different risk of MOF between treatment with GLP-1RA and DPP-4i. We conclude that GLP-1RA are safe in terms of fracture.

## Introduction

Although bone mineral density (BMD) is normal or even elevated in individuals with type 2 diabetes mellitus (T2D), T2D has been associated with an increased fracture risk (1). In addition to increased BMD, individuals with T2D tend to have a higher body mass index (BMI) than controls, which is believed to be protective against fractures (2–4).

Glucagon-like peptide-1 receptor agonists (GLP-1 RA) and dipeptidyl peptidase 4 inhibitors (DPP-4i) were both introduced in Denmark in 2007, and new drugs in the classes are continually being introduced (5). GLP-1 RAs have recently been recommended for treatment of T2D in subjects with cardiovascular disease (6) and are also used for weight loss (7). Consequently, the use of these agents is increasing, creating a need for information on potential effects on other organs such as bone.

Knowledge about the impact of GLP-1 RAs on bone health and fracture risk is limited. Studies attempting to investigate the effects of various glucose-lowering drugs on fracture risk are often subject to confounding and insufficient follow-up durations (8). Cohort studies (9, 10) and meta-analyses (11, 12) have reported GLP-1 RAs to be associated with neutral effects on fracture risk. One meta-analysis, however, found a reduced fracture risk with GLP-1 RAs (13). However, the RCTs analyzed suffer from use of different comparators and short follow-up durations (median durations between 12 weeks and 2 years), and any beneficial effects on fracture rates on such short time-scales may be due to a lower risk of falling rather than improved bone quality. A recent network meta-analysis of 117 RCTs contained estimates of the risk ratios of six separate GLP-1 RAs compared to seven separate DPP-4 inhibitors; findings were neutral except all comparisons against trelagliptin and the comparison of semaglutide to saxagliptin, all of which showed protective effects of the GLP-1 RAs in question (14). All comparisons of GLP-1 RAs to placebo in the network meta-analysis similarly revealed neutral effects except for albiglutide which showed a significant protective effect.

For DPP-4is, most studies reported no association with fracture risk (15–25). However, a few studies did find DPP-4is to be associated with a reduced risk of fractures compared to non-DPP-4i use (26, 27) or compared to glitazones (20).

In the present study, we aimed to investigate fracture risk in individuals using GLP-1 RAs versus individuals using DPP-4is. We hypothesized that there is no difference in fracture risk between the two drug classes.

## Study design and methods

The STROBE guideline for reporting of observational studies was followed (STROBE checklist can be found in **Supplemental Table S1**) (28).

### Study design and setting

We conducted a nationwide registry-based cohort study using data from the Danish national registries. We included all individuals who initiated a combination of metformin and GLP-1 RA or metformin and DPP-4i treatment between January 1^st^ 2007 and December 31^st^ 2018. As subjects were included when either treatment combination was initiated, any previous use of metformin, GLP-1 RA or DPP-4is alone or in combination with any other glucose-lowering drug was allowed. We chose to collect data from 2007 onwards as both GLP-1 RAs and DPP-4is became available in Denmark in 2007. Outcome information was collected by identifying all fracture-related diagnoses from index data onwards. Users of GLP-1 RAs were considered the exposure group, and controls (DPP-4i users) were matched 1:1 using propensity scores.

### Data sources

All data were provided in anonymized form by Statistics Denmark (*Danmarks Statistik*, project identifier no. 703382). Statistics Denmark obtained data from national Danish registries. All Danish citizens are assigned a unique 10-digit personal identification number (PIN) stored in the Danish Civil Registration System, which contains high-fidelity individual-level information on all residents in Denmark and Greenland (29). This PIN allows easy and unambiguous individual-level record linkage between different Danish registers (30, 31). The Danish Government provides full health care to all Danish citizens, including free access to hospitals and full or partial reimbursement of drug expenses. The Danish National Prescription Registry contains information on all prescription drugs sold in Denmark since 1995 according to the Anatomical Therapeutical Chemical (ATC) classification (32, 33). All diagnosis codes are stored in the Danish National Patient Registry, which covers all in- and outpatient contacts to the hospital (34). All physician-assigned discharge diagnoses are included, coded according to the *International Classification of Diseases, Eight Edition* (ICD-8) from 1977 until 1993 and according to ICD-10 from 1994 onwards.

All data on sex, date of birth, death, emigration, and socioeconomic factors were obtained from the Danish Civil Registration System.

### Study population

**Figure 1:**
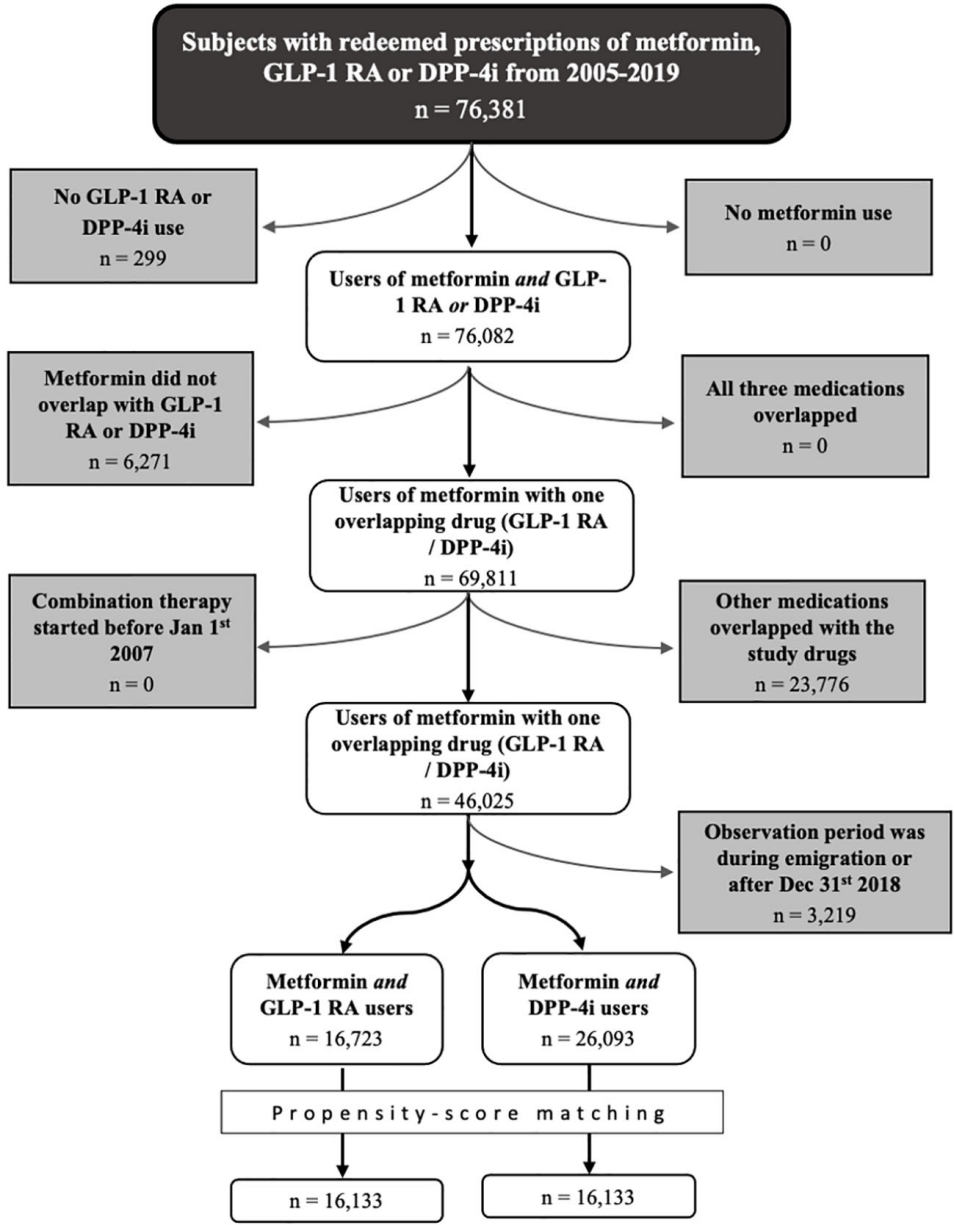
Flowchart of the process of in-/exclusion. DPP-4i, dipeptidyl peptidase 4 inhibitor; GLP-1 RA, glucagon-like peptide-1 receptor agonists.

The study population included subjects residing in Denmark. A flowchart of the inclusion process is presented in **Figure 1**.

We first identified persons treated with metformin and GLP-1 RAs (the exposure drug) and/or DPP-4is (the control drug) between January 1^st^ 2005 and December 31^st^ 2019. These dates were set outside the study period to ensure that follow-up wasn’t initiated inappropriately late or terminated early simply due to natural intervals between redemptions (e.g., an individual with a prescription redemption in Jan 2019 mistakenly has follow-up terminated in early December 2018). For each medication, we defined a start date (date of first redemption) and an end date (date of last redemption plus the number of daily doses redeemed on that date). We then excluded all individuals in which treatment with GLP-1 RAs and DPP-4is overlapped for the entire duration of treatment and those in which neither medication overlapped with metformin use. Remaining individuals were assigned to the exposure or control group based on which medication was first taken singularly in combination with metformin.

Then start and end dates were defined for each other class of glucose-lowering medication. Those who were already treated with an additional glucose-lowering drug (or several) at the beginning of combination therapy were included if (and when) the additional medication was halted and the individual thus received only a combination of metformin and GLP-1 RA or metformin and DPP-4i treatment. *End of combination therapy* was defined as the day that treatment with metformin, the exposure drug, or the control drug ceased or when another glucose-lowering drug was initiated. Glucose-lowering drugs were defined as any drugs with an ATC code beginning in “A10”; i.e., insulins and analogues, biguanides, sulfonylureas, alpha-glucosidase inhibitors, thiazolidinediones, DPP-4is, GLP-1 RAs, sodium-glucose co-transporter 2 inhibitors, and repaglinide.

Finally, the cohort was limited to those in which *beginning of combination therapy* was between January 1^st^ 2007 and Dec 31^st^ 2018.

### Exposure

The National Prescription Registry contains data on redeemed drug prescriptions along with dates, doses and pack sizes. Each medication–including the exposure and control medications–was only considered used if an individual had redeemed at least three prescriptions in the period outlined above. Medications were identified using ATC codes (**Supplemental Table S2**).

From the National Prescription Registry, we obtained the Defined Daily Doses (DDD) variable, which is based on “the assumed average maintenance dose per day for a drug used for its main indication in adults”, according to the World Health Organization Collaborating Centre for Drug Statistics Methodology (35). The resultant number of days was added to the date of last prescription redemption to estimate a true end-of-treatment for each drug.

Of note, exposure to metformin, the exposure drug, and the control drug was assumed to be continuous between initiation and end-of-treatment. To estimate the effects of pauses in these drugs, we calculated the cumulative dose (total number of DDDs) for each drug between the last prescription redeemed prior to or at index date until end of follow-up for each individual. We then assessed pauses using the medication possession ratio (MPR); the ratio of the cumulative number of daily doses to the number of days in the same period. To remove the effects of pauses in medication or low average medication dose, several thresholds for MPR were used: MPR ≥ 0.5, MPR ≥ 0.75, and MPR ≥ 0.95. Lower thresholds likely exclude individuals without pauses in medication, whereas higher thresholds more likely relate to the actual dosage that individuals receive (i.e., those with no pauses but receiving low-to-intermediate doses are excluded with these thresholds).

The follow-up period was defined as the time between the index date and *end of combination therapy*, emigration, death, or December 31^st^ 2018, whichever came first.

### Outcomes

The primary outcome was incident major osteoporotic fracture (MOF). MOF were defined as any of the following fractures: Hip, vertebral, humerus, or forearm fracture. Fractures were identified by ICD-10 codes (**Supplemental Table S3**). The risks of any fracture, hip fracture, vertebral fracture, humerus fracture, and forearm fracture were estimated in secondary analyses.

### Covariates

Data on covariates were obtained using ICD-8 (1977–1993) and ICD-10 (1993–2018) codes (**Supplemental Table S2**), ATC codes (1995–2018) (**Supplemental Table S3**), or a combination of both (**Supplemental Table S4**). All covariates were assessed at baseline (index date) and did not vary over time.

Age at baseline was calculated from the index date and date of birth. Debut of diabetes was estimated as first-ever prescription for glucose-lowering drug, and diabetes duration at baseline was calculated as the time between diabetes debut until index date.

Osteoporosis was defined as the presence of diagnosis codes for osteoporosis, previous/current treatment with antiosteoporotic medications and/or previous MOF; the variable was assigned three levels (2 = previous MOF, 1 = treatment/diagnosis, 0 = none).

As a proxy for heavy smoking (binary variable), we used diagnosis codes related to lung diseases highly associated with tobacco exposure along with diagnosis codes related to nicotine or tobacco, previous use of medications for the treatment of tobacco dependence, and initiation of drugs for obstructive airway disease after the age of 40.

Obesity, alcohol consumption and hypertension (binary variables) were defined by any diagnosis codes related to the conditions in question and/or ever use of medications for their treatment.

Late-diabetic complications, inflammatory bowel disease (IBD), kidney disease, and previous falls (binary variables) were identified through diagnosis codes.

The Charlson Comorbidity Index (CCI, numeric variable) was calculated based on other comorbidities. The CCI was modified to exclude kidney disease and late-diabetic complications, as these covariates were separately adjusted for in the statistical analyses.

Previous insulin use and previous glucocorticoid use were identified through redeemed prescriptions (binary variables).

Income (numeric variable) along with marital status and employment status (categorical variables; the latter classified by Statistics Denmark according to the so-called *SOCIO13 classification*) were identified on the year preceding each individual’s index year. Income (in Danish Kroner, DKK) was adjusted for inflation to a 2018 level according to the Consumer Price Index provided by Statistics Denmark and converted from DKK to Euros using an exchange rate of 7.4363 DKK/Euro.

## Statistical analysis

### Descriptive statistics

Descriptive statistics are presented as numbers and proportions (%), means and standard deviations (SD), or medians and interquartile ranges (IQR). In the case of CCI, median and 10^th^-90^th^ percentile were presented rather than median and IQR, as we expected a large majority of all subjects to have CCI values of 0 or 1. Standardized mean differences (SMD) were also calculated for all baseline variables as recommended for propensity-score matched studies (36). Cohen suggested that SMD values above 0.2 be considered small, SMD values above 0.5 considered medium-sized, and SMD values above 0.8 considered large (36, 37).

### Missing data

There were only missing data in the socioeconomic variables (marital status, income, and employment). Income was used as a covariate in the main analysis, and missing data were imputed beforehand. Missing data were assumed to be missing at random, and multiple imputation was performed by multivariate imputation using chained equations (38, 39). Ten imputations were produced, each of which ran for ten iterations. As the proportion of missing data was very low (0.3%), and the covariate (income) appeared to be balanced between groups and not alter the results of the survival analysis, it was omitted from all subgroup and sensitivity analyses.

### Propensity-score matching

Due to imbalances in sex, age at baseline, and inclusion date, we matched the two groups on propensity scores estimated from these variables. A binomial logistic model was fitted to age, sex, and inclusion date using treatment group as the dependent variable (40, 41) and propensity scores were predicted for each individual in the main cohort.

We matched subjects 1:1 on the logit transformation of the propensity score by nearest-neighbor (“greedy”) matching without replacement, using a caliper width equal to 0.2 x the (pooled) SD of the transformed propensity scores (42, 43).

For multiple imputed datasets, matching and statistical analyses were performed separately on each resultant dataset, and the statistical estimates were finally pooled.

For subgroups, matching was done using the previously computed propensity scores. In the subgroups examining specific GLP-1 RAs, *k*:1 matching was performed, with *k* being the highest possible number up to 10 which allowed every individual in the exposure group to be matched to *k* controls within the set calipers.

After matching, balance in the matched variables was assessed by inspecting the distributions of propensity scores across groups and by calculating SMDs for each matching variable.

### Multicollinearity

Multicollinearity was assessed using the Variance Inflation Factor (VIF) which yielded values no higher than 1.4 for any covariate. In addition, we examined Pearson’s partial correlation coefficient for each pair of variables, and none revealed significant correlations.

### Survival analysis

On a non-imputed matched dataset, the Kaplan-Meier Estimator was used to produce survival plots for all fracture types; a survival plot for MOF on a non-matched dataset was also produced (44). For each subgroup and sensitivity analysis, Kaplan-Meier curves for MOF were also produced.

For the primary analysis, we used the Cox proportional hazards model to estimate hazard rate ratios (HRs) for fracture between the exposure and the control groups. We estimated both crude and adjusted HRs for primary and secondary outcomes. The proportional hazards assumption was evaluated by examining the scaled Schoenfeld residuals of each variable (45). In the fully adjusted model, the covariate osteoporosis was found to violate the proportional hazards assumption and was therefore used as a stratification variable rather than included in the adjustment model. To account for pairing in the matched dataset, a robust variance estimator was used (46, 47).

Finally, to examine a possible additive effect of GLP-1 RAs on fracture risk, we used Aalen’s additive hazards regression model; that is, to examine whether absolute rather than relative differences in hazard could be found (48). In short, Aalen’s additive hazards model produces a plot for each included covariate, depicting how the given covariate affects the absolute hazard of the outcome at all timepoints; i.e., all effects are allowed to be time-varying. The plot for the intercept corresponds to the baseline hazard that an individual would experience if effects from all covariates and exposure were set to zero.

### Sensitivity and subgroup analyses

Several sensitivity and subgroup analyses were performed.

First, we examined males and females separately. Second, we performed sensitivity analyses excluding those with low MPR (selected thresholds are described previously in the section Exposure) in either metformin or study drug (GLP-1 RA or DPP-4i) during the study period. Third, we examined a cohort excluding individuals with kidney disease, previous pancreatitis, or previous falls. Fourth, we performed a sensitivity analyses excluding individuals with follow-up times less than 6 months. Fifth, we split the GLP-1 RA group into specific drug groups–liraglutide, semaglutide, exenatide, dulaglutide, and lixisenatide–based on the drug of which they had received the largest cumulative dose during the study period; ties were handled by allowing any person to appear in several of these subgroups, and only three persons did so. Sixth, we performed the main analysis in the full cohort without prior matching. Seventh, we performed a sensitivity analysis excluding individuals treated with systemic glucocorticoids within the last year prior to inclusion, while not allowing follow-up to continue past initiation of systemic glucocorticoid treatment. Lastly, we performed an analysis analogous to the “intention-to-treat” approach in clinical trials; we continued follow-up after changes in medication for an extra 2 years – or until death or emigration, whichever came first. The sensitivity and subgroup analyses were performed on matched groups unless stated otherwise.

### Statistical software

All analyses were performed using R 4.1.0 (The R Core Team & The R Foundation for Statistical Computing, Vienna, Austria) in the integrated development environment (IDE) RStudio 1.4.1106 (RStudio, PBC, Boston, MA, USA). For imputation, the package “mice” (v 3.13.0) was used. Matching was performed using “MatchIt” (v. 4.2.0) and, for multiply imputed datasets, “MatchThem” (v. 1.0.0). Survival analyses–i.e., Cox model, Kaplan-Meier estimator, and Aalen’s additive hazards regression–were performed using packages “Survival” (v. 2.1.11), “Survminer” (v. 0.4.9), and Survey (v. 4.0).

## Results

### Baseline characteristics

We identified 42,816 subjects treated with metformin in combination with either GLP-1 RAs (n = 16,723) or DPP-4is (n = 26,093). After propensity-score matching, a total of 32,266 (16,133 in each group) remained.

**Table 1** shows baseline characteristics of subjects in either group in both the full cohort and the matched cohort. The most noticeable differences between the unmatched GLP-1 RA group and the DPP-4i group were sex (43.1% vs. 40.3% females, respectively), age (mean 56.6 vs. 63.6 years, respectively), income (median 35,458 vs. 30,459 euros, respectively), and employment status (59.0% vs. 41.1% retired, respectively). Upon matching, these differences were highly attenuated, and matching was satisfactory. Data from the matched cohort will be presented in short in the following.

**Table 1:** Baseline characteristics of full and matched cohorts.

	Full Cohort		Matched Cohort		
	GLP-1 RA group	DPP-4i group	GLP-1 RA group	DPP-4i group	SMD
**n =**	16,723	26,093	16,133	16,133	
**Sex (female),** n (%)	7,210 (43.1%)	10,510 (40.3%)	6,827 (42.3%)	6,660 (41.3%)	0.021
**Age (years),** mean (±SD)	56.6 (±12.0)	63.6 (±12.4)	57.5 (±11.3)	57.9 (±11.0)	0.034
**Follow-up time (days),** median [IQR]	637 [222–1,403]	519 [196–1,133]	642 [223–1,414]	529 [207–1,131]	0.157
**Inclusion Year,** n (%)					**0.290**
2007	23 (0.1%)	712 (2.7%)	22 (0.1%)	428 (2.7%)	
2008	171 (1.0%)	1,639 (6.3%)	160 (1.0%)	1,035 (6.4%)	
2009	439 (2.6%)	1,207 (4.6%)	421 (2.6%)	777 (4.8%)	
2010	2,026 (12.1%)	1,752 (6.7%)	1,986 (12.3%)	1,130 (7.0%)	
2011	2,397 (14.3%)	2,074 (7.9%)	2,313 (14.3%)	1,276 (7.9%)	
2012	2,107 (12.6%)	2,047 (7.8%)	2,045 (12.7%)	1,204 (7.5%)	
2013	1,544 (9.2%)	2,270(8.7%)	1,488 (9.2%)	1,416 (8.8%)	
2014	1,290 (7.7%)	2,598 (10.0%)	1,232 (7.6%)	1,585 (9.8%)	
2015	1,446 (8.6%)	2,887 (11.1%)	1,384 (8.6%)	1,846 (11.4%)	
2016	1,457 (8.7%)	3,128 (12.0%)	1,394 (8.6%)	1,841 (11.4%)	
2017	1,607 (9.6%)	2,986 (11.4%)	1,559 (9.7%)	1,824 (11.3%)	
2018	2,216 (13.3%)	2,793 (10.7%)	2,129 (13.2%)	1,771 (11.0%)	
**Diabetes Duration (years),** median [IQR]	4.84 [2.07–8.44]	4.51 [1.71–5.54]	4.95 [2.15–8.55]	3.80 [1.33–7.03]	**0.240**
**Charlson Comorbidity Index,** mean (±SD)	0.69 (±1.12]	0.92 (±1.34)	0.70 (±1.13)	0.73 (±1.20)	0.023
**Charlson Comorbidity Index,** n (%)					0.024
Score 0	10,066 (60.2%)	13,947 (53.5%)	9,624 (59.7%)	9,655 (59.8%)	
Score 1	3,779 (22.6%)	5,873 (22.5%)	3,675 (22.8%)	3,539 (21.9%)	
Score 2	1,742 (10.4%)	3429 (13.1%)	1,708 (10.6%)	1,687 (10.5%)	
Score 3	683 (4.1%)	1,555 (6.0%)	678 (4.2%)	731 (4.5%)	
Score ≥4	450 (2.7%)	1,289 (4.9%)	448 (2.8%)	521 (3.2%)	
**Complications of diabetes,** n (%)	4,275 (25.6%)	5,545 (21.3%)	4,188 (26.0%)	2,936 (18.2%)	0.188
Diabetic Neuropathy	694 (4.2%)	884 (3.4%)	690 (4.3%)	423 (2.6%)	0.091
Diabetic Nephropathy	499 (3.0%)	787 (3.0%)	489 (3.0%)	393 (2.4%)	0.036
Diabetic Retinopathy	1,192 (7.1%)	1,375 (5.3%)	1,160 (7.2%)	784 (4.9%)	0.098
Other	2,968 (17.7%)	3,787 (14.5%)	2,912 (18.1%)	1,947 (12.1%)	0.168
**Osteoporosis,** n (%)					0.031
No history	14,851 (88.8%)	22,504 (86.2%)	14,338 (88.9%)	14,272 (88.5%)	
Diagnosed / Treated	244 (1.5%)	687 (2.6%)	242 (1.5%)	307 (1.9%)	
Previous MOF	1,628 (9.7%)	2,902 (11.1%)	1,553 (9.6%)	1,554 (9.6%)	
**Risk factors for falls,** n (%)					
Hypoglycemic episodes	145 (0.9%)	368 (1.4%)	136 (0.8%)	150 (0.9%)	0.009
Previous Falls	669 (4.0%)	1080 (4.1%)	645 (4.0%)	584 (3.6%)	0.020
Visual Impairment	180 (1.1%)	442 (1.7%)	178 (1.1%)	188 (1.2%)	0.006
**Any pancreatitis,** n (%)	289 (1.7%)	514 (2.0%)	281 (1.7%)	306 (1.9%)	0.012
Acute Pancreatitis	261 (1.0%)	450 (1.7%)	253 (1.6%)	276 (1.7%)	0.011
Chronic Pancreatitis	63 (0.4%)	158 (0.6%)	61 (0.4%)	93 (0.6%)	0.029
**Glucose-lowering drug use (prior to study period),** n (%)					
Metformin	16,377 (97.9%)	25,340 (97.1%)	15,807 (98.0%)	15,664 (97.1%)	0.057
SGLT2 inhibitors	694 (4.2%)	380 (1.5%)	673 (4.2%)	239 (1.5%)	0.163
GLP-1 receptor agonists	4,540 (27.1%)	178 (0.7%)	4,463 (27.7%)	124 (0.8%)	**0.835**
DDP-4 inhibitors	1,157 (6.9%)	4,242 (16.3%)	1,131 (7.0%)	2,222 (13.8%)	**0.223**
Insulin, any	2,256 (13.5%)	1,261 (4.8%)	2,156 (13.4%)	793 (4.9%)	**0.296**
Sulfonylureas	6,277 (37.5%)	8,248 (31.6%)	6,194 (38.4%)	4,279 (26.5%)	**0.256**
Alpha-glucosidase inhibitors	112 (0.7%)	136 (0.5%)	111 (0.7%)	61 (0.4%)	0.043
Glitazones	807 (4.8%)	1,033 (4.0%)	797 (4.9%)	572 (3.5%)	0.069
Repaglinide	337 (2.0%)	404 (1.5%)	336 (2.1%)	199 (1.2%)	0.067
**Hypertension,** n (%)	13,303 (79.5%)	21,046 (80.7%)	13,054 (80.9%)	12,168 (75.4%)	0.133
**Chronic Kidney Disease,** n (%)	545 (3.3%)	1,263 (4.8%)	533 (3.3%)	599 (3.7%)	0.022
**Liver Disease,** n (%)	488 (2.9%)	762 (2.9%)	472 (2.9%)	494 (3.1%)	0.008
Mild	447 (2.7%)	664 (2.5%)	431 (2.7%)	438 (2.7%)	0.003
Moderate to severe	84 (0.5%)	175 (0.7%)	84 (0.5%)	105 (0.7%)	0.017
**Hyperparathyroidism,** n (%)	84 (0.5%)	149 (0.6%)	84 (0.5%)	82 (0.5%)	0.002
**Hyperthyroidism,** n (%)	453 (2.7%)	897 (3.4%)	443 (2.7%)	442 (2.7%)	0
**Hypogonadism,** n (%)	36 (0.2%)	41 (0.2%)	36 (0.2%)	31 (0.2%)	0.007
**Eating disorder or malabsorption,** n (%)	82 (0.5%)	230 (0.9%)	72 (0.4%)	116 (0.7%)	0.036
**Venous thromboembolism,** n (%)	1,419 (8.5%)	2,316 (8.9%)	1,403 (8.7%)	1,258 (7.8%)	0.033
**Inflammatory bowel disease,** n (%)	532 (3.2%)	900 (3.4%)	505 (3.1%)	541 (3.4%)	0.013
**Osteoarthritis,** n (%)	2,804 (16.8%)	4,518 (17.3%)	2,785 (17.3%)	2,261 (14.0%)	0.090
**Dementia,** n (%)	931 (5.6%)	1,813 (6.9%)	888 (5.5%)	973 (6.0%)	0.023
**Alcohol,** n (%)	1,178 (7.0%)	1,862 (7.1%)	1,153 (7.1%)	1,251 (7.8%)	0.023
**Smoking,** n (%)	5,572 (33.3%)	8.699 (33.3%)	5,519 (34.2%)	4,933 (30.6%)	0.078
**Obesity,** n (%)	6,929 (41.4%)	6,058 (23.2%)	6,708 (41.6%)	4,284 (26.6%)	**0.321**
**Other medications (prior to study period),** n (%)					
Statins	13,229 (79.1%)	20,664 (79.2%)	12,959 (80.3%)	12,385 (76.8%)	0.087
Thiazides	7,306 (43.7%)	11,756 (45.1%)	7,219 (44.7%)	6,241 (38.7%)	0.123
Loop Diuretics	4,294 (25.7%)	7,019 (26.9%)	4,245 (26.3%)	3,344 (20.7%)	0.132
Potassium-saving diuretics	2,100 (12.6%)	3,480 (13.3%)	2,071 (12.8%)	1,731 (10.7%)	0.065
Antipsychotics drugs	2,160 (12.9%)	3,240 (12.4%)	2,047 (12.7%)	2,221 (13.8%)	0.032
Antiepileptics drugs	2,485 (14.9%)	3,546 (13.6%)	2,388 (14.8%)	2,255 (14.0%)	0.023
Antiarrhythmic drugs	299 (1.8%)	541 (2.1%)	297 (1.8%)	218 (1.4%)	0.039
Hypnotics	5,091 (30.4%)	7,892 (30.2%)	4,965 (30.8%)	4,578 (28.4%)	0.053
Antidepressants	6,431 (38.5%)	8,740 (33.5%)	6,177 (38.3%)	5,665 (35.1%)	0.066
Anxiolytics	4,914 (29.4%)	7,739 (29.7%)	4,797 (29.7%)	4,591 (28.5%)	0.028
Opioids	9,651 (57.7%)	14,437 (55.3%)	9,400 (58.3%)	8,582 (53.2%)	0.102
NSAID	14,911 (89.2%)	22,448 (86.0%)	14,430 (89.4%)	13,921 (86.3%)	0.097
Sex hormones	5,297 (31.7%)	6,612 (25.3%)	4,950 (30.7%)	4,417 (27.4%)	0.073
Antacids	8,794 (52.6%)	13,710 (52.5%)	8,251 (52.8%)	8,147 (50.5%)	0.046
Glucocorticoids	5,560 (33.2%)	8607 (33.0%)	5,436 (33.7%)	4,977 (30.8%)	0.061
**Income (euros),** median [IQR]	35,458 [25,456–51,287]	30,459 [23,026–44,975]	*35,613* *[25,512–51,563]*	*34,162* *[25,067–49,448]*	*0.038*
**Income quintiles,** n (%)					*0.066*
1^st^	2,772 (16.6%)	5,779 (22.1%)	*2,631 (16.3%)*	*2,772 (17.2%)*	
2^nd^	2,931 (17.5%)	5,607 (21.5%)	*2,850 (17.7%)*	*2,980 (18.5%)*	
3^rd^	3,255 (19.5%)	5,291 (20.3%)	*3,114 (19.3%)*	*3,284 (20.4%)*	
4^th^	3,683 (22.0%)	4,869 (18.7%)	*3,537 (21.9%)*	*3,526 (21.9%)*	
5^th^	4,056 (24.3%)	4,497 (17.2%)	*3,977 (24.7%)*	*3,526 (21.9%)*	
Missing Data	26 (0.2%)	50 (0.2%)	*24 (0.1 %)*	*45 (0.3%)*	
**Marital Status,** n (%)					0.027
Unmarried	3,274 (19.6%)	3,822 (14.8%)	2,935 (18.2%)	3,097 (19.2%)	
Married / Registered Partnership	9,568 (57.2%)	14,867 (57.0%)	9,365 (58.0%)	9,304 (57.7%)	
Divorced / Annulled Partnership	2,756 (16.5%)	4,002 (15.3%)	2,711 (16.8%)	2,550 (15.8%)	
Widowed	1,054 (6.3%)	3,309 (12.7%)	1,054 (6.5%)	1,105 (6.8%)	
Missing Data	71 (0.4%)	93 (0.4%)	68 (0.4%)	77 (0.5%)	
**Employment status,** n (%)					0.033
Working	7,882 (47.1%)	8,800 (33.7%)	7,588 (47.0%)	7,395 (45.8%)	
Unemployed	1,462 (8.7%)	1,380 (5.3%)	1,322 (8.2%)	1,249 (7.7%)	
Retired	6,878 (41.1%)	15,406 (59.0%)	6,795 (42.1%)	7,052 (43.7%)	
Student	131 (0.8%)	58 (0.2%)	78 (0.5%)	57 (0.4%)	
Other	344 (2.1%)	399 (1.5%)	326 (2.0%)	335 (2.1%)	
Missing Data	26 (0.2%)	50 (0.2%)	24 (0.1%)	45 (0.3%)	

Alle data are presented as n (%); mean (±SD); or median [IQR]. DPP-4i, dipeptidyl peptidase 4 inhibitor; GLP-1 RA, glucagon-like peptide-1 receptor agonists; SGLT2, sodium-glucose co-transporter 2; SMD, standardized mean difference; MOF, major osteoporotic fractures; NSAID, non-steroid anti-inflammatory drugs. SMDs above 0.2 are highlighted with bold font. Data on income in the matched cohort (italicized) are presented without imputations.

Median [IQR] follow-up times in the two groups were of 642 [223–1,414] days in the GLP-1 RA group and 529 [207–1,131] days in the DPP-4i group. In total, we had 75,848 years of combined follow-up time.

Sex was balanced between the groups with 42.3% females in the GLP-1 RA group vs. 41.3% in the DPP-4i group. The GLP-1 RA group had a mean ( ± SD) age of 57.5 ( ± 11.3) vs. 57.9 ( ± 11.0) years in the DPP-4i group. Median [IQR] diabetes duration was longer in the GLP-1 RA group with 4.95 [2.15–8.55] years compared to 3.80 [1.33–7.03] years in the DPP-4i group. CCI scores were balanced with medians [10^th^-90^th^ percentile] of 0 [0–2] in both groups. Previous MOF were equally prevalent (9.6%) in both groups.

Subjects in the GLP-1 RA group had more complications of diabetes (26.0% vs. 18.2%) and a higher occurrence of hypertension (80.9% vs. 75.4%) compared to the DPP-4i group, although these differences were below the minimum SMD threshold of 0.2. In addition, those in the GLP-1 RA group were more likely to have a history of obesity (41.6% vs. 26.6%, SMD 0.321) and had a slightly larger fraction of subjects included in the years 2010-2012 and 2018 and a smaller fraction included in the years 2007-2009 compared to the DPP-4i group. The only covariates with SMDs above the minimum threshold of 0.2 were inclusion year, diabetes duration, obesity, and previous use of DPP-4is, GLP-1 RAs, insulins, and sulfonylureas; with previous use of GLP-1 RAs exhibiting an SMD of 0.835. In short, GLP-1 RA users had longer diabetes duration, higher prevalence of obesity, and higher prevalence of previous use of insulins and sulfonylureas than those in the DPP-4i group.

Socioeconomic variables were balanced between groups.

### Risk of major osteoporotic fractures

**Figure 2:**
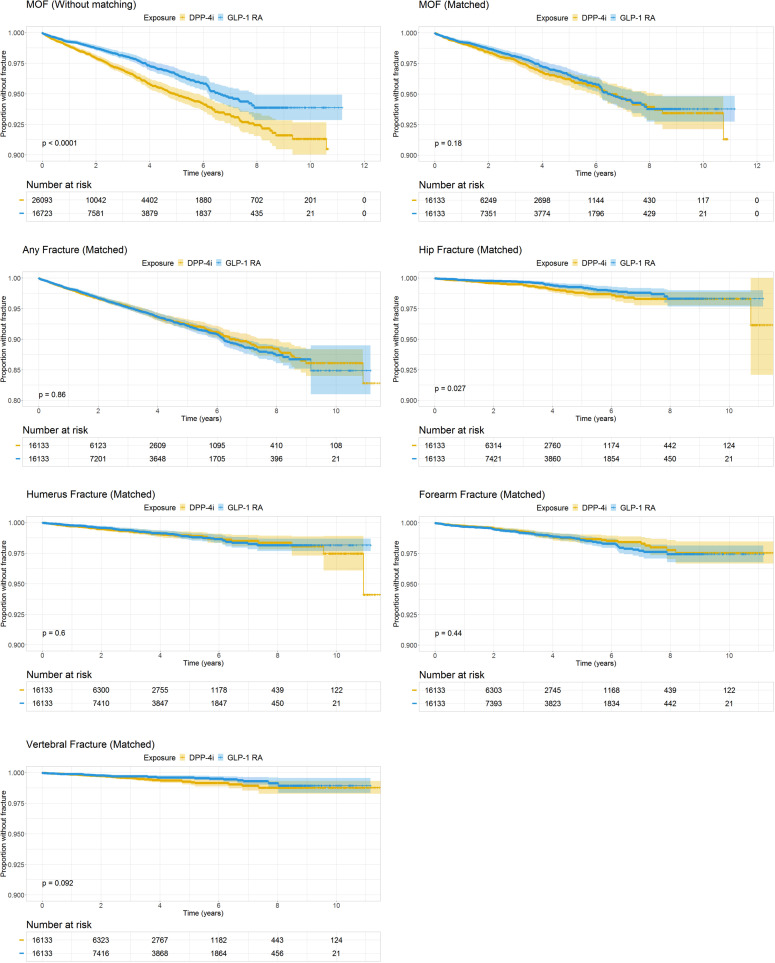
Kaplan-Meier Survival Curves of fracture. Survival curves are presented with *number-at-risk* tables. Time in years on the x-axes. Note, the y-axes go from 0.80 or 0.90 to 1.00. DPP-4i, dipeptidyl peptidase 4 inhibitor; GLP-1 RA, glucagon-like peptide-1 receptor agonists; MOF, Major osteoporotic fracture.

**Table 2** presents HRs for fractures in the matched cohort during the study period. A MOF occurred in 1.8% (n = 286) and 1.7% (n = 274) of GLP-1 RA users and DPP-4i users, respectively. The Crude HR for MOF with GLP-1 RAs compared to DPP-4is was 0.89 [0.76–1.05]. When adjusted for age and sex, this did not change (HR 0.91 [0.77–1.07]), nor did the fully adjusted model alter the result (HR 0.86 [0.73–1.03]). For each analysis in **Table 2** and for the unmatched analysis of MOF, we also present Kaplan-Meier survival curves for crude illustrations (**Figure 2**), which yielded non-significant results in all analyses of the matched cohort.

**Table 2:** Hazard Ratios (HR) for various fracture types in the matched cohort.

Fracture	Fractures, n (%)	Unadjusted (HR [95% CI])	Age & sex (HR [95% CI])	Full model (HR [95% CI])
**MOF**	GLP-1 RA: 286 (1.8)	0.89 [0.76 – 1.06]	0.91 [0.77 – 1.07]	0.86 [0.73 – 1.03]
	DPP-4i: 274 (1.7)			
**Any**	GLP-1 RA: 647 (4.0)	1.01 [0.90 – 1.13]	1.01 [0.90 – 1.13]	0.97 [0.86 – 1.09]
	DPP-4i: 552 (3.4)			
**Hip**	GLP-1 RA: 61 (0.4)	**0.68 [0.49 – 0.96]**	0.71 [0.51 – 1.00]	**0.65 [0.46 – 0.93]**
	DPP-4i: 75 (0.5)			
**Vertebral**	GLP-1 RA: 40 (0.2)	0.70 [0.46 – 1.07]	0.72 [0.47 – 1.10]	0.71 [0.46 – 1.11]
	DPP-4i: 49 (0.3)			
**Humerus**	GLP-1 RA: 89 (0.6)	0.92 [0.68 – 1.24]	0.93 [0.69 – 1.26]	0.91 [0.66 – 1.25]
	DPP-4i: 84 (0.5)			
**Forearm**	GLP-1 RA: 116 (0.7)	1.12 [0.85 – 1.47]	1.10 [0.84 – 1.46]	1.06 [0.79 – 1.41]
	DPP-4i: 88 (0.5)			

DPP-4i, dipeptidyl peptidase 4 inhibitor; GLP-1 RA, glucagon-like peptide-1 receptor agonists; HR, Hazard Ratio; MOF, major osteoporotic fracture; Bold font: the HR was significantly different from 1.00.

Full model: Adjusted for sex, age, inclusion date, diabetes duration, Charlson Comorbidity Index, any diabetic complication, previous falls, inflammatory bowel disease, ever insulin use, ever glucocorticoid use, hypertension, kidney disease, alcohol, smoking, obesity and income, and stratified by osteoporosis.

We found similar results when estimating HRs for hip, vertebral, and humerus fractures, although only hip fractures yielded a significant protective effect of GLP-1 RAs; the crude HR for hip fracture with GLP-1 RAs compared to DPP-4is was 0.68 [0.49–0.96], which was unaltered in the fully adjusted model (HR 0.65 [0.46–0.93]). The crude HR for vertebral fractures was 0.70 [0.46–1.07] with no change after full adjustment (HR 0.71 [0.46–1.11]) when comparing GLP-1 RAs with DPP-4is. For the humerus, the crude HR was 0.92 [0.68–1.24], and the adjusted HR was 0.91 [0.66–1.25] when comparing GLP-1 RAs with DPP-4is. Estimates for any fracture and for forearm fractures were neutral; for forearm fracture the crude HR was 1.12 [0.85–1.47] and the fully adjusted HR 1.06 [0.79–1.41], and for any fracture the crude HR was 1.01 [0.90–1.13], and the fully adjusted HR was 0.97 [0.86-1.09] when comparing GLP-1 RAs with DPP-4is.

### Subgroup and sensitivity analyses

Subgroup and sensitivity analyses are presented in **Table 3**. For the various analyses, Kaplan-Meier curves are presented in **Supplemental Figure S1**.

**Table 3:** Hazard Ratios for MOF in subgroup and sensitivity analyses.

Analysis	n =	Fractures, n (%)	Unadjusted (HR [95% CI])	Age & sex (HR [95% CI])	Full model (HR [95% CI])
**Males**	GLP-1 RA: 9,409	103 (1.1)	0.90 [0.68 – 1.18]	0.90 [0.69 – 1.18]	0.85 [0.64 – 1.12]
	DPP-4i: 9,409	101 (1.1)			
**Females**	GLP-1 RA: 6,622	184 (2.8)	0.96 [0.78 – 1.19]	1.00 [0.80 – 1.23]	0.97 [0.77 – 1.22]
	DPP-4i: 6,622	156 (2.4)			
**MPR ≥ 0.5**	GLP-1 RA: 12,897	222 (1.7)	0.90 [0.75 – 1.09]	0.92 [0.76 – 1.11]	0.87 [0.71 – 1.06]
	DPP-4i: 12,897	211 (1.6)			
**MPR ≥ 0.75**	GLP-1 RA: 9,590	152 (1.6)	**0.80 [0.64–0.998]**	0.81 [0.64 – 1.01]	**0.75 [0.60 – 0.95]**
	DPP-4i: 9,590	163 (1.7)			
**MPR ≥ 0.95**	GLP-1 RA: 6,195	83 (1.3)	**0.72 [0.54 – 0.97]**	**0.73 [0.54 – 0.96]**	**0.62 [0.46 – 0.84]**
	DPP-4i: 6,195	99 (1.6)			
**No CKD etc.**	GLP-1 RA: 14,726	251 (1.7)	0.88 [0.74 – 1.05]	0.90 [0.75 – 1.07]	0.85 [0.70 – 1.02]
	DPP-4i: 14,726	244 (1.7)			
**6+ months follow-up**	GLP-1 RA: 12,695	275 (2.2)	0.86 [0.73 – 1.02]	0.88 [0.74 – 1.04]	0.84 [0.71 – 1.00]
	DPP-4i: 12,695	274 (2.2)			
**Liraglutide**	GLP-1 RA: 14,961	280 (1.9)	0.92 [0.77 – 1.09]	0.93 [0.79 – 1.11]	0.89 [0.75 – 1.06]
	DPP-4i: 14,961	249 (1.7)			
**Semaglutide**	GLP-1 RA: 615	1 (0.2)	0.81 [0.11 – 5.98]	N/A	N/A
	DPP-4i: 4,305	71 (1.6)			
**Exenatide**	GLP-1 RA: 435	3 (0.7)	0.42 [0.13 – 1.34]	N/A	N/A
	DPP-4i: 3,480	52 (1.5)			
**Dulaglutide**	GLP-1 RA: 325	3 (0.9)	1.25 [0.32 – 4.91]	N/A	N/A
	DPP-4i: 975	13 (1.3)			
**Lixisenatide**	GLP-1 RA: 15	0 (0)	N/A	N/A	N/A
	DPP-4i: 150	2 (1.3)			
**Full cohort (unmatched)**	GLP-1 RA: 16,723	290 (1.7)	**0.67 [0.58 – 0.78]**	0.91 [0.78 – 1.05]	0.87 [0.74 – 1.02]
	DPP-4i: 26,093	578 (2.2)			
**Glucocorticoid as exclusion**	GLP-1 RA: 14,635	242 (1.7)	0.93 [0.78 – 1.12]	0.95 [0.79 – 1.14]	0.89 [0.74 – 1.08]
	DPP-4i: 14,635	219 (1.5)			
**Intention-to-treat analysis**	GLP-1 RA: 16,133	410 (2.5)	0.89 [0.78 – 1.02]	0.90 [0.79 – 1.03]	**0.85 [0.74 – 0.98]**
	DPP-4i: 16,133	425 (2.6)			

DPP-4i, dipeptidyl peptidase 4 inhibitor; GLP-1 RA, glucagon-like peptide-1 receptor agonists; HR, Hazard Ratio; MOF, major osteoporotic fracture; MPR, medication possession rate; N/A, not available. Bold font = the HR was significantly different from 1.00.

“No pause”: excluded those with pauses in metformin, SGLT2 inhibitor or GLP-1 receptor agonist during the study period. “No CKD etc.”: Excluded those with chronic kidney disease, previous falls and previous chronic pancreatitis. “6+ months follow-up”: Excluding all with follow-up times less than 183 days.

Full model: Adjusted for sex, age, inclusion date, diabetes duration, Charlson Comorbidity Index, any diabetic complications, previous falls, inflammatory bowel disease, ever insulin use, ever glucocorticoid use, hypertension, kidney disease, alcohol, smoking and obesity, and stratified by osteoporosis.

Effects between groups were similar between males and females. No changes in effect sizes were observed when excluding individuals with chronic kidney disease, previous pancreatitis and previous falls. When examining different thresholds for MPR, a clear trend was apparent with larger difference in fracture risk for increasing MPR thresholds between the GLP-1 RA group and the DPP-4i group. At MPR ≥ 0.5, the adjusted HR was quite similar to that in the main analysis (HR 0.87 [0.71-1.06]), but this became lower at MPR ≥ 0.75 (HR 0.75 [0.60 – 0.95]) and lower yet at MPR ≥ 0.95 (HR 0.62 [0.46 – 0.84])

When excluding subjects with follow-up times shorter than 6 months, 25,390 individuals remained, and the unadjusted HR for MOF was found to be 0.86 [0.73–1.02] for GLP-1 RAs compared to DPP-4is. The fully adjusted model yielded a similar HR of 0.84 [0.71–1.00].

Dividing the GLP-1 RA group into subgroups based on the specific drug yielded five groups; liraglutide, semaglutide, exenatide, dulaglutide, and lixisenatide. However, liraglutide users comprised the far majority of GLP-1 RA users (92%), and no other subgroup had sufficient fracture rates to allow reasonable estimation of HRs.

Examining the full (unmatched) cohort for MOF risk yielded a significant protective effect in the GLP-1 RA group (unadjusted HR 0.67 [0.58–0.78]) compared to the DPP-4i group. The same effect can be seen in the Kaplan-Meier plot of MOF in the unmatched cohort (p < 0.0001). However, this effect was attenuated in the fully adjusted model to entirely resemble the matched analyses (HR 0.87 [0.74–1.02]).

The results were not altered when defining recent or ongoing glucocorticoid use as an exclusion criterion (adjusted HR for MOF 0.89 [0.74–1.08]). When performing an “intention-to-treat” analysis, the HR for MOF was found to be 0.89 [0.78–1.02] with GLP-1 RAs compared to DPP-4is, although this became significant in the fully adjusted model (HR 0.85 [0.74 – 0.98]).

**Figure 3:**
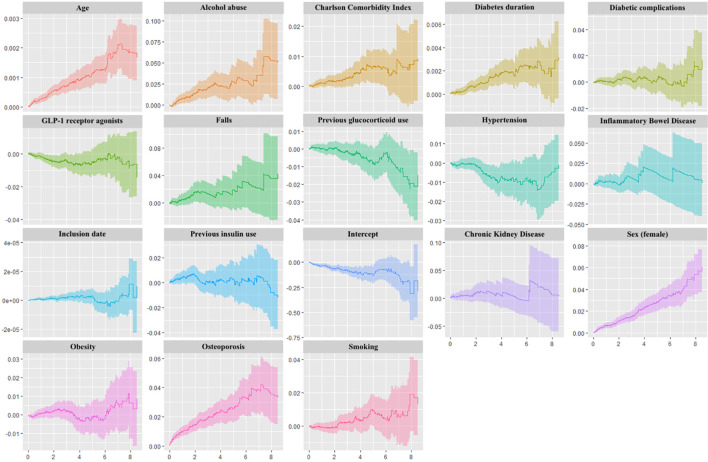
Aalen’s Additive Regression Plots. Plots of the time-varying additive hazards plotted against time (years) on the x-axes for covariates used in Aalen’s Additive Regression Model. This regression model assumes additive risks (producing hazard rate differences) rather than multiplicative risks (producing hazard rate ratios) for each covariate. The plots contain the cumulative hazards attributable to each covariate, and the slope at any point on the plot corresponds to a hazard rate; positive slopes represent increased risks, and negative slopes represent reduced risks. Note that effects may be time-varying, and the slopes can therefore be positive at one timepoint and negative at another timepoint. The intercept term represents a baseline hazard (the hazard of an individual for whom exposure and all covariate values are zero).

Using Aalen’s additive hazards regression model, we attempted to model the effects of the drugs on fracture in an entirely different way (**Figure 3**). This test revealed a near-significant protective effect of the GLP-1 RAs compared to DPP-4is with a slope of -0.0042 (p = 0.051). However, this slope only reflects a linear approximation to the time-varying effect of the analysis. Assessing the plot, the excess hazard was initially negative (significantly so), but temporarily increased towards zero after around four to six years of exposure, after which it declined once more; this is consistent with a protective effect of GLP-1 RAs on both short and long time-scales. Performing the Aalen’s additive hazards model on the *intention-to-treat* analysis revealed a continuing downward slope for GLP-1 RAs and a less pronounced attenuation on intermediate time-scales (slope -0.0038, p = 0.015).

As a final measure, we analyzed deaths in the two groups in order to assess potential bias induced by an imbalance in these. In the GLP-1 RA group, 190 (1.2%) deaths occurred with a median [IQR] time-to-event of 647 [188–1,407] days, whereas the DPP-4i group experienced 175 (1.1%) deaths with a median time-to-event of 549 [230–1099] days. Indeed, the crude HR for death (with MOF as a censoring event) in the GLP-1 RA group with the DPP-4i group as reference was 0.94 [0.77–1.15]. When adjusted for age and sex, this became 0.96 [0.78–1.18] and when fully adjusted 0.78 [0.64–0.98].

In addition to the estimates of treatment on fracture risk, we have presented all covariate estimates from the main analysis of MOF in **Supplemental Table S5**. Please note that these are merely associations as they appear in the given model and do not represent effects that may be interpreted in any causal manner.

## Discussion

### Summary of findings

In the present study, we found that the risk of MOF was slightly lower, albeit not significantly, in those treated with GLP-1 RA compared to those treated with DPP-4is as add-on therapies to metformin. HRs were generally on the order of magnitude of 0.85-0.90; i.e., a 10-15% lower risk of fractures with GLP-1 RAs. These results were similar across various analyses, which will be summarized in the following.

Examining specific fracture sites revealed non-significantly reduced risk of fractures of the humerus and of the spine with GLP-1 RAs compared to DPP-4is. Interestingly, however, in the case of hip fractures, we found a statistically significant effect of GLP-1 RAs compared to DPP-4i with risk reductions of as much as 30-35%. Risks of any fracture and of forearm fracture were similar between the groups.

When estimating HRs for MOF in the full unmatched cohort, the unadjusted analysis yielded a highly significant difference between the groups. However, this higher risk in the unmatched DPP-4i group appeared to be confounded by age, as the unmatched DPP-4i group was on average 7 years older than the GLP-1 RA group; indeed, the effect was attenuated in the adjusted analyses to resemble the results of the main analysis.

In our “intention-to-treat” sensitivity analysis with an additional two years of follow-up, we found an unadjusted HR very similar to the main analysis, although this became a significant protective effect in the fully adjusted analysis. Since changes in bone tissue manifest as fractures with a long delay, this may hint at more pronounced slow-acting effects on bone of the two drugs, although imbalances in confounding factors between the groups may also arise as time passes.

Similarly, when increasing the minimum thresholds for average daily dose received in the analysis of MOF, differences between the two groups became larger and increasingly significant. Although this study was not designed to examine a dose-response relationship between the exposure and the outcome, this finding may indicate a dose-dependent effect. This lends credence to a causal interpretation of the associations discussed above.

As increased fall risk may be a contributor to the fracture risk in diabetes (49), we performed a subgroup analysis excluding those with known previous falls. In addition, we adjusted for covariates related to falls, diabetic neuropathy, diabetic retinopathy, and visual impairment.

In order to rule out differential mortality as a source of bias in our study, we estimated HRs of death in the two groups and found a negligible difference, although this became significant in the fully adjusted model. A lower mortality in the GLP-1 RA group would expectedly lead to an underestimation of the fracture risk in that group, thereby exaggerating a protective effect of GLP-1 RAs. However, due to the small number of deaths, we believe that the magnitude of such an effect must be negligible.

### Previous research

Observational studies and meta-analyses of RCTs on fracture risk with GLP-1 RAs have found mostly neutral effects (9–12, 14, 50), although one meta-analysis found reduced risk of fractures (13). Similarly, studies on DPP-4is have found neutral effects on fracture risk (23–25). Most studies, however, are limited by short follow-up durations (8). Furthermore, research on glucose-lowering drugs and fracture risk is subject to much heterogeneity between studies, particularly due to the many different choices of comparators. Performing randomized controlled clinical trials on the timescales required for long-term outcomes as osteoporotic fractures is often not feasible in a general population not otherwise at high risk of fractures.

However, studies on markers of bone health point towards direct beneficial effects of GLP-1 RAs on bone. Two randomized controlled trials demonstrated reduced bone loss during weight loss with GLP-1 RA compared to placebo (51, 52). Indeed, osteoblastic cell lines express GLP-1 receptors (53), and GLP-1 receptor knockout mice exhibited increased bone resorption and cortical osteopenia (54).

### Strengths and limitations

This cohort study was performed using data from Danish nationwide registries. These contain individual-level data on all prescription medications and diagnosis codes along with socioeconomic factors. This provides high-fidelity information on diseases and treatments in the whole period in which GLP-1 RAs and DPP-4is have been marketed in Denmark, allowing an unbiased study population with very little missing data, and providing results which are highly generalizable to other similar populations.

The use of DPP-4is as a comparator provided a highly comparable control group, particularly as both drugs were used in the setting of sole add-on medication to metformin, and both drugs have similar priority in the management of T2D. However, GLP-1 RAs are often preferred for T2D subjects with obesity or cardiovascular disease, providing a potential for confounding by indication. Although we attempted to adjust for this, we did not have direct measurements of BMI. In addition, a large proportion of the GLP-1 RA group (13.8%) had received DPP-4is before baseline, whereas only 0.8% of the DPP-4i group had received GLP-1 RAs prior. This indicates that DPP-4i treatment is in some cases attempted before switch to GLP-1 RAs, as the cost of DPP-4is is lower, and GLP-1 RAs (during the period in which this study was conducted) required injections. The price difference between the drugs was reflected in the income gap between the two groups, which was however diminished with matching. The tendency for some individuals to have received DPP-4is before switching to GLP-1 RAs may account for the longer diabetes duration and the slightly higher prevalence of diabetic complications and hypertension in the GLP-1 RA group.

Propensity-score matching is a method of mimicking some of the characteristics of a randomized controlled trial (41, 42), and it provided us with fairly balanced matching. However, matching resulted in the discarding of many subjects; the cohort reduced from 42,816 to 32,266 individuals. To examine whether this introduced any bias or resulted in the loss of efficiency, a sensitivity analysis was performed on the full cohort.

In addition, a variety of subgroup and sensitivity analyses confirmed the finding from the main analysis. This supports the conclusion of neutral or slightly reduced risk of fracture with GLP-1 RA treatment compared to DPP-4i treatment in this population.

Residual confounding in an observational study cannot be ruled out. Particularly, we were unable to account for diet and exercise, both of which may serve as confounders. Lack of access to lab results and other clinical information prevented adjustment for variables such as BMD, BMI, and glycemic control (e.g., HbA1c). In addition, some covariates such as smoking and alcohol consumption were crudely estimated through diagnosis codes and previous medications. Similarly, the utility of diagnosis codes to identify falls and other risk factors for fracture is limited, and therefore differential fall patterns between the two groups may still be a cause of residual confounding. However, those treated with GLP-1 RAs appear to have higher prevalence of late-diabetic complications and previous SU and insulin use. These factors indicate that the GLP-1 RA group is more severely affected by diabetes compared to the DPP-4i group, and the GLP-1 RA group may thus be subject to residual confounding associated with higher fracture risk; this would in turn lead to over-estimation of fracture risk in those receiving GLP-1 RAs. As a consequence, the true HR would potentially be more in favor of GLP-1 RAs than the HRs observed in this study.

As changes in bone structure take time to manifest as fractures, median follow-up times less than 2 years may not be sufficient to fully assess the effects of these drugs. However, in the matched cohort, a full 13,767 individuals had more than two years of follow-up time, with nearly half of those (n = 6,650) having more than four years.

## Conclusion

In our primary analysis, the risk of MOF was not significantly different between users of GLP-1 RA and DPP-4i. However, in a secondary analysis, users of GLP-1 RA exhibited a significantly lower risk of hip fracture and a lower risk of MOF compared to DPP-4i users when allowing follow-up to continue after medication change. In addition, when examining higher doses of treatment, the difference in MOF risk between the two groups became increasingly larger (with increasing statistical significance) with higher dose thresholds. In contrast, the remaining analyses of MOF revealed fracture risks that are comparable between DPP-4i users and GLP-1 RA users. The results of this study are in line with previous research and support the continued use of GLP-1 RAs in the management of T2D in patients at risk of fracture.

## Data availability statement

The datasets presented in this article are not readily available because. All eligible research organizations can apply for access to data at Statistics Denmark. Requests to access the datasets should be directed to https://www.dst.dk/da/TilSalg/Forskningsservice/Dataadgang.

## Author contributions

All authors contributed to the article according to the ICJME requirements for co-authorship. All authors critically revised the paper for intellectual content and approved the submitted versions and the final version of the manuscript. ZKA, RV, and JS-L designed the study. ZKA, RV, JS-L and PV had access to all data used in the study. ZKA performed data management and statistical analyses with assistance from all co-authors. ZKA interpreted the data and wrote the manuscript. JS-L, RV, PV, RF-N and SG made ongoing critical revisions of study design and data interpretation.

## Funding

This work was supported by a Steno Collaborative Grant, Novo Nordisk Foundation, Denmark (Grant no. NNF18OC0052064).

## Conflict of interest

The authors declare that the research was conducted in the absence of any commercial or financial relationships that could be construed as a potential conflict of interest.

## Publisher’s note

All claims expressed in this article are solely those of the authors and do not necessarily represent those of their affiliated organizations, or those of the publisher, the editors and the reviewers. Any product that may be evaluated in this article, or claim that may be made by its manufacturer, is not guaranteed or endorsed by the publisher.
